# Between Hope and Skepticism: Parent and Provider Expectations of Artificial Intelligence as a Bridge to Human‐Centered Care for Children With Chronic Illness

**DOI:** 10.1111/hex.70705

**Published:** 2026-06-03

**Authors:** Atefeh Shamsi, Mahboobeh Namnabati, Asghar Ehteshami, Hamed Zandi Esfahani

**Affiliations:** ^1^ Nursing Care Research Center, Clinical Sciences Institute, Faculty of Nursing, Baqiyatallah University of Medical Sciences Tehran Iran; ^2^ Nursing and Midwifery Care Research Center, Isfahan University of Medical Sciences Isfahan Iran; ^3^ Health Information Technology Research Center, Isfahan University of Medical Sciences Isfahan Iran; ^4^ Department of Pediatrics School of Medicine, Emam Hossein Hospital Research Center, Isfahan University of Medical Sciences Isfahan Iran

**Keywords:** artificial intelligence, caregivers, chronic disease, health services accessibility, qualitative research, rural health services, telemedicine

## Abstract

**Background:**

Parents of children with chronic diseases in rural Iran experience profound challenges, including limited access to pediatric specialists, social isolation, and severe financial strain. Concurrently, healthcare providers face workforce shortages, administrative burdens, and fragmented referral systems. This study aimed to explore unmet care needs and comparative perspectives of parents and providers regarding the integration of artificial intelligence to strengthen chronic disease management in resource‐limited rural settings.

**Methods:**

Using a qualitative descriptive design, we conducted semi‐structured interviews with 20 parents of children with chronic illnesses and 15 healthcare providers (physicians, nurses, and community health workers) from rural health centres in Iran. Participants were selected through purposive sampling based on direct experience with pediatric chronic disease management in villages (< 20,000 population). Data were analysed using conventional content analysis with iterative coding to derive emergent themes.

**Results:**

Five key themes emerged: (1) Unmet daily care needs including geographic barriers to specialists, maternal emotional isolation, and catastrophic out‐of‐pocket expenses; (2) Systemic constraints faced by providers, notably administrative overload (“pajama time”), critical workforce shortages, and inefficient referral pathways; (3) AI as a potential bridge through symptom prediction models for early intervention, chatbots for emergency guidance, and AI‐enabled teleconsultations to reduce unnecessary travel; (4) Divergent trust narratives parents expressed skepticism about autonomous AI decision‐making while providers raised concerns about data privacy, workload implications, and erosion of clinical authority; and (5) Integration pathways emphasising AI embedded within the existing Behvarz (community health worker) network, mandatory digital literacy training, and co‐designed platforms incorporating local cultural beliefs.

**Conclusion:**

AI technologies show promise for augmenting, though not replacing, human‐centred care in rural pediatric chronic disease management. Successful implementation requires culturally resonant, transparent tools developed through participatory design with families and providers, robust data governance, and strategic alignment with Iran's primary healthcare infrastructure. This context‐sensitive framework prioritises equity, trust‐building, and caregiver empowerment while acknowledging the irreplaceable role of human empathy in chronic care delivery.

**Patient or Public Contribution:**

Parents of children with chronic illnesses and healthcare providers were central to this research as knowledge partners rather than passive subjects. Twenty parents with lived experience of caring for a child with chronic disease in rural settings, alongside 15 frontline healthcare providers, actively shaped the study through in‐depth sharing of their experiences during semi‐structured interviews. Their narratives directly informed all emergent themes and the resulting conceptual framework. To ensure interpretive validity, we conducted member checking with a purposive subset of participants (*n* = 8 parents and *n* = 6 providers) who reviewed preliminary findings and provided feedback on whether the themes accurately reflected their realities and concerns. This iterative validation process strengthened the trustworthiness of our analysis and ensured that the final framework resonated with the everyday challenges and aspirations of rural families and providers. While participants were not involved in the initial study design or manuscript drafting due to the exploratory nature of this qualitative investigation, their experiential expertise fundamentally shaped the research outcomes and recommendations for culturally grounded AI integration. Their contributions transformed abstract technological possibilities into contextually meaningful pathways for supporting rural pediatric chronic disease management.

**Abbreviations**
AI: artificial intelligence
CHW: community health worker
COREQ: Consolidated Criteria for Reporting Qualitative Research
EMR: electronic medical record
HCP: healthcare provider
IF: impact factor
LMIC: low‐ and middle‐income country
NVivo: qualitative data analysis software
N/A: not applicable
PHC: primary health care
STS: socio‐technical systems
TAM: technology acceptance model
UAE: United Arab Emirates
WHO: World Health Organization

## 1 Introduction/Background

The management of chronic illness in children represents a significant challenge for healthcare systems worldwide, requiring ongoing support, monitoring, and coordination between families and healthcare providers. In resource‐limited settings, such as rural communities in Iran, these challenges are amplified by geographic barriers, limited access to specialist services, and workforce shortages. The emergence of artificial intelligence (AI) technologies has generated considerable optimism about their potential to address these healthcare access gaps, yet the implementation of AI in low‐ and middle‐income countries remains understudied, particularly in the context of pediatric care.

The existing literature on AI in chronic disease management has predominantly focused on high‐income countries with robust digital infrastructure. Systematic reviews by top journals have documented the potential of AI to improve diagnostic accuracy, enhance treatment adherence, and facilitate remote monitoring [1, 2, 3]. However, these studies have largely been conducted in settings where healthcare infrastructure, internet connectivity, and digital literacy are substantially higher than in resource‐limited contexts. A systematic review by Wang et al. [4] identified 47 studies examining AI acceptance in healthcare, yet only three (6.4%) were conducted in low‐ and middle‐income countries, and none specifically addressed pediatric chronic illness care [5]. This significant gap in the literature leaves critical questions unanswered about how AI might be perceived and adopted in settings where healthcare resources are constrained.

Furthermore, existing qualitative investigations have primarily examined AI perceptions among physicians and hospital administrators [6, 7, 8], with minimal attention to community health workers and family caregivers' key stakeholders whose acceptance and engagement are critical for successful AI integration. While technology acceptance models have been extensively validated in Western contexts [9, 10, 11], their applicability to rural healthcare settings with distinct socio‐cultural dynamics remains underexplored. The intersection of AI, pediatric chronic illness, and rural healthcare access in the Middle East represents an especially understudied domain, despite significant investments in digital health infrastructure across the region.

Iran presents a unique context for examining AI adoption in rural healthcare. The country has made substantial progress in expanding telecommunications infrastructure, with internet penetration rates increasing significantly over the past decade. According to Iran's Ministry of Information and Communications Technology (2024), approximately 78% of rural households now have access to mobile internet services, though connectivity speeds and reliability vary considerably across regions [12]. The National Internet Development Plan aims to achieve 95% broadband coverage in rural areas by 2027, yet current infrastructure limitations including inconsistent speeds, bandwidth constraints, and power supply interruptions continue to affect the feasibility and quality of digital health services in remote communities [13].

Central toIran's rural healthcare system is the Behvarz (plural: Behvarzan) community health workers who serve as the primary link between rural communities and the formal healthcare system. Behvarzan undergoes 2–3 years of specialised training in primary healthcare delivery, including maternal and child health, disease prevention, health education, and basic medical care [14]. Each Behvarz serves approximately 1500–2500 residents in rural areas, conducting home visits, health education sessions, and basic diagnostic services. They refer patients to higher‐level facilities when specialised care is required. The Behvarz system has been recognised by the World Health Organization as a successful model of community health worker implementation in middle‐income countries [15]. In the context of pediatric chronic illness, Behvarzan plays a critical role in ongoing monitoring, medication adherence support, and coordination between families and specialist services.

Despite Iran's investments in digital health infrastructure, healthcare providers in rural areas have expressed caution about unconditionally embracing AI. Concerns have been raised about the safety of unreliable tools, the potential for overdiagnosis, and the risk of eroding the doctor‐patient relationship [16]. Providers are particularly concerned about the pressure of unrealistic patient expectations and the adversarial dynamics that can arise when patients present with AI‐generated diagnoses. In contrast, patients and caregivers often perceive AI as a supportive tool that should be taken more seriously in clinical settings where they may feel unheard [17]. This divergence in perception highlights the urgent need for research examining how these two stakeholder groups perceive the role of AI in pediatric chronic illness care.

For this study, we define AI as computer‐based systems designed to perform tasks that typically require human intelligence, including but not limited to [1]: machine learning algorithms that analyze health data to generate predictive insights [2]; natural language processing applications such as conversational chatbots for patient engagement [3]; teleconsultation platforms that facilitate remote clinical decision‐making; and [5] decision support systems that assist healthcare providers in diagnosis and treatment planning [18]. This conceptual boundary was established to ensure consistent understanding among participants and to focus the inquiry on AI applications relevant to pediatric chronic illness care in rural settings.

While the role of AI in chronic disease management has been widely discussed in global literature, research on its application in rural communities with limited resources particularly examining the lived experiences of parents alongside the perspectives of healthcare providers remains sparse. The existing literature lacks [1]: empirical studies from Middle Eastern contexts [2]; comparative analyses of parent and provider perspectives [3]; attention to the role of community health workers in AI implementation; and [5] an understanding of how AI might function as a complement to rather than a replacement of human care in resource‐limited settings.

This study addresses these gaps by exploring the expectations, hopes, and concerns of both parents of children with chronic illnesses and healthcare providers (including Behvarzan, physicians, and nurses) regarding AI as a bridge to human‐centred care in rural Iran. The study focuses on children with diverse chronic conditions, including Type 1 diabetes, asthma, epilepsy, congenital heart disease, chronic kidney disease, sickle cell disease, and cerebral palsy. By employing conventional qualitative content analysis grounded in the perspectives of these key stakeholders, this research contributes empirical evidence to inform culturally appropriate AI implementation strategies in resource‐limited settings.

The specific objectives of this study are:

To explore parents' expectations of AI technologies in managing their children's chronic illnesses in rural Iran.

To examine healthcare providers' (Behvarzan, physicians, nurses) expectations of AI as a tool for pediatric chronic illness care.

To identify factors influencing acceptance and resistance to AI adoption in rural healthcare settings.

To develop empirically grounded recommendations for AI implementation that complement the role of Behvarzan and enhance human‐centred care.

## 2 Methods

### 2.1 Study Design

This study utilised a qualitative conventional content analysis approach, which is an inductive method for deriving codes, categories, and themes directly from textual data without imposing pre‐existing theoretical frameworks. Following the methodology outlined by Hsieh and Shannon [19], this approach allows key concepts and insights to emerge organically from the participants' own words, making it particularly well‐suited for exploring a phenomenon like caregiving and technology adoption from the perspectives of those experiencing it [1]. The study was conducted and reported in accordance with the Consolidated Criteria for Reporting Qualitative Research (COREQ) checklist [19, 20].

### 2.2 Research Team and Setting

The research team consisted of four researchers with diverse expertise in qualitative research and health services: two health services researchers, a qualitative methodologist, and a pediatrician with expertise in rural health. This multidisciplinary composition enhanced the interpretive depth and clinical relevance of the analysis. The first author conducted the majority of interviews, with assistance from a research assistant who held a master's degree in public health. Both researchers received formal training in semi‐structured interviewing techniques before data collection and had prior experience in qualitative health research. The researchers had no prior relationship with participants before the study commencement.

#### 2.2.1 Setting

The study was conducted in three rural districts of Isfahan Province, Iran: Najafabad, Shahin Shahr, and Mobarakeh. These districts were selected to represent diverse geographic and socioeconomic contexts within rural Iran, including variations in proximity to urban specialist services and healthcare infrastructure. The setting was chosen because [1]: these areas have functioning rural health centres (Behvarz posts) staffed by community health workers [2]; there is documented variation in internet connectivity and digital infrastructure; and [3] the population includes families with children affected by various chronic illnesses who receive care through the Behvarz system.

### 2.3 Participants

This study selected two distinct groups of participants from rural areas using purposive sampling with a maximum variation strategy. This method was chosen to ensure the inclusion of individuals who have direct, lived experience of the phenomena under study and to capture a wide range of perspectives.

#### 2.3.1 Parent Participants

Parents were defined as the primary caregivers of children with chronic illnesses residing in rural areas. The inclusion criteria were [1]: primary caregiver residing in a village with a population of less than 20,000 [2]; child diagnosed with a chronic illness (duration ≥ 6 months) [3]; caregiver aged 18 years or older; and [5] willingness to participate in a recorded interview. Non‐primary caregivers were excluded from the study.

#### 2.3.2 Healthcare Provider Participants

Healthcare providers included physicians, nurses, and Behvarzan (community health workers) who served rural populations. The inclusion criteria were [1]: employment in a rural health facility or regular provision of care to a rural population [2]; minimum 2 years of professional experience [3]; patient population including children with chronic illnesses; and [5] willingness to participate in a recorded interview. Administrative staff or city‐based professionals who did not have direct contact with rural families were excluded (Table 1).

**Table 1:** Participant characteristics.

Characteristic	Parents (*n* = 20)	Providers (*n* = 15)
Age, years		
Mean	37.4	41.2
Range	28–52	24–58
Gender, *n* (%)		
Female	14 (70.0)	9 (60.0)
Male	6 (30.0)	6 (40.0)
Education, years		
Mean	11.3	17.8
Range	5–16	14–22
Years in role		
Mean	N/A	14.6
Range	N/A	2–32
Chronic illnesses represented, *n* (%)		
Type 1 diabetes	6 (30.0)	—
Asthma	5 (25.0)	—
Epilepsy	3 (15.0)	—
Congenital heart disease	2 (10.0)	—
Chronic kidney disease	2 (10.0)	—
Sickle cell disease	1 (5.0)	—
Cerebral palsy	1 (5.0)	—
Provider types, *n* (%)		
Behvarz/community health worker	—	10 (66.7)
Physician/pediatrician/family medicine	—	3 (20.0)
Nurse	—	2 (13.3)

*Note:* N/A = not applicable. — = not relevant to the corresponding participant group. Percentages may not total exactly 100.0 because of rounding.

### 2.4 Sample Size Justification and Saturation

The final sample comprised 35 participants: 20 parents and 15 healthcare providers. Sample size was determined based on the principle of theoretical saturation, guided by the criteria outlined by Guest, Bunce, and Johnson and Morse [21, 22].

#### 2.4.1 Saturation Monitoring Approach

We employed a systematic saturation monitoring approach with the following indicators:

No new codes emerging from consecutive interviews within each participant category.

Redundancy of themes across participant groups.

Adequacy of data to support theme development and conceptual mapping.

Sufficient variation in perspectives to ensure representativeness of the phenomenon.

#### 2.4.2 Data Collection Phases and Sample Adequacy

Data collection proceeded in three phases:

1. **Initial sampling (*n* = 20):** Participants were recruited to capture maximum variation across caregiver experiences, child chronic illness types, and provider roles.

2. **Theoretical sampling (*n* = 10):** Additional participants were recruited to explore emerging categories in greater depth, clarify variation between parent and provider perspectives, and examine areas where early analysis suggested incomplete conceptual development.

3. **Confirmation sampling (*n* = 5):** The final interviews were used to assess the stability and completeness of the emerging themes and to determine whether additional interviews generated substantively new codes, categories, or interpretive insights.

We did not conduct a statistical power analysis because this was a qualitative study and was not designed to test hypotheses or estimate effect sizes. Instead, sample adequacy was assessed through purposive maximum‐variation sampling, ongoing saturation monitoring, and the concept of information power. Information power suggests that the required sample size depends on the study aim, sample specificity, quality of interview dialogue, use of theory, and analytic strategy rather than on a predetermined numerical threshold [21, 22, 23, 24].

In the present study, the sample was considered adequate because participants had direct and specific experience with pediatric chronic illness care in rural settings, the study aim was focused, interviews generated rich and relevant accounts, and the analysis was conducted iteratively alongside data collection. Saturation was monitored throughout data collection by documenting the emergence of new codes after each interview and discussing theme development during regular team meetings. By the 30th interview, no new major codes or categories were identified within either participant group. The final five interviews did not generate new themes; rather, they confirmed the stability of the existing thematic structure and added illustrative variation within already established categories, particularly around digital literacy, human oversight, and trust in AI‐supported care.

Thus, the final sample of 35 participants, including 20 parents and 15 healthcare providers, was judged sufficient to address the study objectives and to support the development of the three overarching themes (Table 2).

**Table 2:** Saturation monitoring across data collection phases.

Phase	Interviews	Purpose	Contribution to saturation
Initial sampling	1–20	Capture broad variation across parents, chronic illness types, and provider roles	Generated initial codes, categories, and candidate themes
Theoretical sampling	21–30	Explore emerging categories and clarify parent‐provider differences	Refined categories and strengthened conceptual boundaries
Confirmation sampling	31–35	Test whether new interviews generated new themes	No new major themes emerged; final interviews confirmed theme stability

### 2.5 Data Collection

#### 2.5.1 Interview Protocol

Semi‐structured, individual interviews were conducted with each participant to elicit rich and detailed narratives about their experiences and perceptions. A semi‐structured interview guide was developed based on the research objectives and reviewed by three experts in qualitative research and health technology adoption [19, 20]. The guide included the following domains:

Current experiences with healthcare access for children with chronic illness.

Understanding and perceptions of AI.

Expectations and hopes for AI in pediatric healthcare.

Concerns and barriers to AI adoption.

Role of Behvarzan in AI‐integrated care.

Recommendations for AI implementation.

Probing questions were used to encourage elaboration and explore unexpected topics that emerged during interviews.

#### 2.5.2 Interview Details

**Table:** 

Parameter	Details
Duration	35–75 min (mean: 52 min)
Mode	Face‐to‐face
Location	Private locations chosen by participants (rural health centres, participants' homes, quiet café settings)
Language	Persian (participants' native language)
Audio recording	With full consent of each participant
Transcription	Verbatim within 48 h of interview
Field notes	Written immediately after each interview

The decision to conduct face‐to‐face interviews was based on the need to build rapport with participants in a culturally appropriate manner and to accommodate participants who had limited familiarity with video conferencing technology.

#### 2.5.3 Data Collection Period

Interviews were conducted between March and August 2024.

### 2.6 Translation Procedure

Given that interviews were conducted in Persian and the manuscript was prepared in English, we implemented a structured cross‐language qualitative translation procedure to preserve conceptual meaning, cultural nuance, and analytic credibility. The translation process was not treated as a purely technical linguistic task, but as an interpretive component of qualitative analysis, consistent with methodological guidance on cross‐language qualitative research.

First, all audio recordings were transcribed verbatim in Persian by the first author and checked against the recordings for accuracy. Initial coding and theme development were conducted in Persian to reduce the risk of premature meaning loss during translation. After the analytic themes had been developed, selected participant quotations and relevant analytic excerpts were translated into English for manuscript preparation.

The translation process involved six steps:

1. Persian transcription and verification: All interviews were transcribed verbatim in Persian. The first author reviewed the transcripts against the audio recordings to ensure accuracy and completeness.
2. Forward translation into English: Selected quotations and analytic excerpts were translated from Persian into English by a bilingual member of the research team with experience in health services research.
3. Independent translation review: A second bilingual researcher, who was not involved in the initial translation, reviewed the English translations against the Persian transcripts to assess conceptual accuracy rather than literal word‐for‐word equivalence.
4. Resolution of discrepancies: Disagreements or ambiguities were discussed by the bilingual members of the research team. Particular attention was paid to idiomatic expressions, culturally embedded meanings, and emotionally significant phrases that did not have direct English equivalents.
5. Translation documentation: The team maintained a translation decision log documenting key translation choices, culturally specific expressions, and terms for which literal translation could distort participants' intended meaning.
6. Final methodological review: The translated excerpts were reviewed by the qualitative methodologist to ensure readability, conceptual consistency, and alignment with the final themes.

This procedure was informed by methodological recommendations for cross‐language qualitative research, particularly the need to document translator roles, preserve conceptual equivalence, and make translation decisions transparent. In line with Squires' guidance, we considered translation as part of the trustworthiness of the study rather than as a mechanical step after analysis. Temple and Young's discussion of translation dilemmas in qualitative research also informed our attention to the interpretive role of translators and the potential influence of translation on meaning construction [25, 26, 27].

### 2.7 Data Analysis

Data analysis followed conventional qualitative content analysis as described by Hsieh and Shannon [19], which is appropriate for studies aiming to describe a phenomenon where existing theory or research literature is limited [1]. This approach allows categories to derive directly from the data through open coding, category development, and abstraction, without imposing pre‐existing theoretical frameworks on the findings.

#### 2.7.1 Analytical Process

The analysis process involved three main stages:

1. Open Coding: Initial codes were generated by reading transcripts line‐by‐line. The first author read all transcripts repeatedly to achieve immersion and develop a holistic understanding of the data. Initial open coding generated 847 initial codes across the dataset.

2. Category Development: Codes were grouped into broader categories based on similarity and relatedness. The research team engaged in collaborative categorisation through regular consensus meetings.

3. Abstraction: Categories were refined and integrated into overarching themes through iterative abstraction, where subcategories were grouped into higher‐order categories (Table 3).

**Table 3:** Example of coding process.

Initial code	Category	Theme
Long travel distances	Geographic barriers	Structural Barriers
Specialist shortage		
Emotional exhaustion	Caregiver burden	
Financial strain		
Early warning systems	Predictive analytics	AI as Bridge
Emergency guidance	Chatbots	
Virtual specialist visits	Teleconsultation	
Need for doctor oversight	Human trust	Trust Dynamics
Data privacy concerns	Provider concerns	
Human connection valued	Cultural values	

#### 2.7.2 Rigor and Trustworthiness

To ensure analytical rigor and trustworthiness, we employed several strategies:

Prolonged engagement with data through repeated reading of transcripts.

Audit trail maintains a detailed record of all analytical decisions.

Peer debriefing sessions with two external qualitative research experts.

Member checking with six participants to validate preliminary findings.

Reflexive journaling to document researcher assumptions and potential biases.

#### 2.7.3 Intercoder Reliability

Two researchers independently coded a subset of six transcripts (approximately 17% of the dataset). Initial intercoder agreement was 78%, which is within the acceptable range for qualitative research [11]. Following discussion and consensus development, agreement increased to 94%. Discrepancies were resolved through a return to the original data and consultation with the qualitative methodologist.

Regular consensus meetings were held throughout the analysis process (biweekly over 6 months), where the full research team discussed emerging codes, resolved interpretive challenges, and refined category definitions. A detailed codebook was developed and iteratively updated, documenting each code's definition, examples, and inclusion/exclusion criteria.

The analysis was conducted using NVivo software (version 14) to facilitate systematic organisation and retrieval of coded data. All Persian‐language analysis (coding and category development) was conducted in Persian to maintain conceptual integrity, with translations to English occurring after theme finalisation.

#### 2.7.4 Ethical Considerations

The study was adhered to all ethical guidelines for research involving human subjects. Informed consent was obtained from all participants, detailing the purpose of the study, the voluntary nature of their participation, and the confidentiality of their responses. Anonymity was ensured by using pseudonyms for all participants and removing any identifying information from the data. The study protocol was approved by the Ethics Committee of Isfahan University of Medical Sciences (Approval code: IR.MUI.NUREMA.REC.2022.055).

#### 2.7.5 Findings

Analysis of the interview transcripts revealed three overarching themes that encompassed the experiences and perceptions of parents and healthcare providers regarding chronic disease management and the potential role of AI in rural healthcare. Rather than presenting findings as isolated categories, this section provides an interpretive narrative that contextualises quotations within the broader analytical framework, following the guidance of Goldberg and Allen [28] on communicating qualitative research effectively.

**Figure 1:**
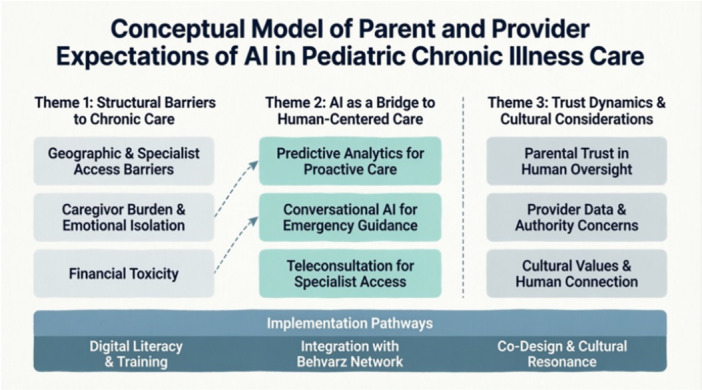
Conceptual model of parent and provider expectations of AI in pediatric chronic illness care.

The thematic structure is illustrated in Figure 1, which depicts the hierarchical relationships among themes, categories, and subcategories. The conceptual model demonstrates how parent and provider expectations are shaped by contextual factors including rural healthcare infrastructure, cultural considerations, and previous experiences with technology adoption.

## 3 The Three Major Themes

The three major themes are:

1. Structural Barriers to Chronic Care—The systemic challenges that both parents and providers face in rural healthcare delivery.

2. AI as a Bridge to Human‐Centred Care—Hopeful anticipations about AI's potential to address healthcare access gaps.

3. Trust Dynamics and Cultural Considerations—The complex factors influencing acceptance and resistance to AI adoption.

### 3.1 Theme 1: Structural Barriers to Chronic Care

The first major theme describes the structural conditions that shaped both parents' caregiving experiences and providers' capacity to deliver care for children with chronic illness in rural settings. Rather than representing isolated difficulties, participants' accounts showed how geographic distance, limited specialist availability, emotional strain, and financial pressure interacted to produce a continuous burden of care. These barriers also shaped how participants later imagined the possible role of AI: not as a technological replacement for care, but as a potential means of reducing delays, improving access to advice, and supporting decision‐making within an already constrained rural health system.

This theme was evident across both parent and provider interviews. Parents primarily described structural barriers through the practical and emotional consequences for daily family life, whereas providers described the same barriers through the lens of service capacity, referral limitations, and the difficulty of managing complex pediatric cases in under‐resourced rural clinics. By the confirmation interviews, no new structural barriers were identified; later accounts mainly reinforced the same categories and added variation in how families and providers experienced them.

#### 3.1.1 Geographic and Specialist Access Barriers

Geographic distance from specialist pediatric care was one of the most consistently described barriers in the dataset. Parents did not describe distance simply as a matter of travel time. Rather, distance was experienced as uncertainty, delayed help‐seeking, disruption to family routines, and fear that a child's condition might worsen before specialist advice could be obtained. For parents, the problem was therefore not only the physical absence of specialists, but also the emotional burden of having to decide when a long and costly journey was necessary.

One mother described this combination of distance, uncertainty, and delayed access:

Every time my son's condition worsens, I face a long and tiring journey to see a specialist who understands his needs. It seems like the nearest specialist is always hours away, and with transportation limitations, timely care often seems impossible.

(Mother, P‐07)

This quotation illustrates how geographic distance was interpreted by parents as a threat to timely care. The phrase “timely care often seems impossible” suggests that the burden was not limited to transportation itself, but included the fear of being unable to act quickly enough during clinical deterioration. Similar concerns appeared in other parent interviews, particularly when families described repeated travel to urban hospitals, uncertainty about whether symptoms required urgent review, and the difficulty of arranging transport while managing other household responsibilities.

Healthcare providers described the same access barrier from a service‐delivery perspective. They emphasised that rural clinics were often expected to manage complex pediatric chronic conditions despite limited specialist availability and constrained referral pathways:

Our rural clinic has a hard time finding a pediatrician. Families can't get the specialized care their children need anywhere nearby, so we struggle to manage complex cases with limited resources.

(Community Health Worker, HP‐03)

The provider account complements the parent quotation by showing that access barriers were not only experienced by families but also recognised within the rural care system. Providers' concern was not merely that specialists were geographically distant, but that the absence of specialist support shifted clinical responsibility onto rural health workers who often lacked the resources needed for complex pediatric chronic disease management.

Taken together, parent and provider accounts indicate convergence around a shared structural problem: specialist care was physically distant, referral pathways were burdensome, and rural clinics had limited capacity to compensate for these gaps. However, the meaning of this barrier differed by participant group. Parents emphasised anxiety, exhaustion, and fear of delayed care, whereas providers emphasised workforce shortages, limited clinical backup, and the pressure of managing cases beyond the ideal capacity of rural facilities.

Evidence of saturation for this subtheme was observed during the later stages of data collection. By the final phase of interviews, participants continued to describe distance, travel burden, and limited specialist availability, but these accounts did not generate new categories. Instead, they confirmed the stability of the subtheme and showed its relevance across different chronic illness types and provider roles. This supported the interpretation of geographic and specialist access barriers as a central structural condition shaping expectation of AI‐supported care.

#### 3.1.2 Caregiver Burden and Emotional Isolation

Caregiver burden emerged as a second dimension of structural constraint, particularly among mothers who described themselves as the primary managers of their child's daily care. Unlike geographic barriers, which were often visible through travel and referral difficulties, emotional isolation was described as a less visible but persistent burden. Parents' accounts suggested that chronic illness caregiving extended into nearly every aspect of daily life, including medication routines, symptom monitoring, financial decisions, household responsibilities, and constant vigilance about possible deterioration.

Mothers in particular described caregiving as morally meaningful but emotionally exhausting. Their narratives did not simply present caregiving as “burden”; rather, they showed an ambivalent experience in which caring for the child was understood as an important parental responsibility while also producing loneliness, fatigue, and a sense of personal loss. One mother explained:

As a mother, I sometimes feel like I'm carrying the weight of the world alone, trapped in a silent room where no one really sees the weariness behind my smile. Caring for my child is sacred, but it often feels like an endless loneliness, as if this burden is taking me away from the life I once knew.

(Mother, P‐09)

This account illustrates the emotional contradiction that appeared across several parent interviews: caregiving was described as deeply valued, but also as isolating when the caregiver's own needs remained unrecognised. The phrase “behind my smile” suggests that distress was often hidden rather than openly expressed, while “the life I once knew” points to perceived changes in identity, social participation, and personal freedom after the child's diagnosis.

Healthcare providers also recognised this hidden emotional burden, although they described it from the perspective of repeated encounters with families in rural clinics. One provider noted:

I've seen too many mothers who, despite being heroes at home, are emotionally isolated, captive to cultural expectations of unwavering devotion. They rarely express their loneliness, yet this loneliness has overshadowed every aspect of their well‐being, silently expressing things that healthcare rarely addresses.

(Healthcare Provider, HP‐04)

The provider's account supports the interpretation that caregiver isolation was not only an individual psychological experience, but also a relational and service‐level issue. Providers were aware that mothers often carried substantial emotional strain, yet routine healthcare encounters appeared to focus primarily on the child's clinical condition rather than the caregiver's well‐being. This gap may help explain why some parents later expressed interest in AI‐supported reminders, remote guidance, or easier access to advice: these tools were not imagined only as technical solutions, but also as ways to reduce the feeling of managing care entirely alone.

Across the dataset, caregiver burden was most strongly articulated by mothers, but providers also confirmed its visibility in clinical practice. Later interviews did not introduce new dimensions of emotional isolation; instead, they reinforced the recurring pattern that caregiving was experienced as continuous, socially underrecognized, and insufficiently addressed within routine rural healthcare. This supported the saturation of the subtheme and its inclusion as a central component of structural barriers to chronic care.

#### 3.1.3 Financial Toxicity in Rural Healthcare

Financial strain was another recurrent dimension of structural burden in rural pediatric chronic care. Parents described the costs of chronic illness not only in terms of direct medical expenses, but also through transportation costs, lost work time, medication costs, and the need to borrow money or sell household resources to reach specialist care. In this sense, financial burden was closely connected to the geographic access barriers described above: distance from specialist services made each episode of care more expensive and more disruptive for families.

For parents, the financial burden of care was experienced as a repeated trade‐off between seeking treatment and preserving the household's limited economic resources. One mother described this dilemma:

Every time my child gets sick, I face a difficult choice: either sell some of our limited produce or borrow money just to pay for the trip to the city hospital. The cost of travel, medicine, and waiting time slowly eats away at what little we have. It's as if illness costs more than health it saps our hope.

(Mother, P‐06)

This quotation illustrates how the cost of care extended beyond the clinic or hospital bill. The reference to selling produce or borrowing money suggests that care‐seeking could directly affect household survival strategies. The phrase “illness costs more than health” captures the cumulative nature of this burden: each episode of illness generated expenses that reduced the family's ability to maintain everyday life, creating a sense that chronic disease management was economically and emotionally draining.

Providers described the same issue from the perspective of delayed or interrupted care. Rather than presenting financial hardship as a private family matter, providers framed it as a factor that influenced whether families could follow referral advice, attend scheduled appointments, or obtain medicines consistently:

I see families who skip scheduled appointments because they simply don't have the money for transportation or medicine, even if the child's condition worsens. Financial pressure creates invisible barriers that no amount of advice can overcome this often means children stay sick longer or end up in emergencies.

(Healthcare Provider, H‐12)

This provider account shows that financial hardship shaped clinical trajectories. Advice to seek care or continue treatment was not always actionable when families lacked money for transport or medication. The phrase “invisible barriers” is analytically important because it shows that non‐attendance or delayed care should not be interpreted simply as lack of awareness, motivation, or adherence. Instead, participants' accounts suggest that financial strain constrained families' ability to act on medical recommendations.

Across parent and provider interviews, financial toxicity was therefore closely linked with delayed care‐seeking, interrupted follow‐up, and increased emotional pressure on caregivers. Parents emphasised sacrifice, borrowing, and loss of household resources, while providers emphasised missed appointments, incomplete treatment, and worsening clinical conditions. These complementary perspectives support the interpretation of financial toxicity as a structural barrier rather than an individual‐level problem.

Saturation for this subtheme was supported by the recurrence of similar financial concerns across the later interviews. During confirmation sampling, participants continued to describe transportation expenses, medication costs, and missed appointments, but these accounts did not generate new categories. Instead, they reinforced the interpretation that financial burden interacted with distance and specialist scarcity to shape families' access to chronic care.

These findings suggest that participants' later interest in AI‐supported teleconsultation and remote guidance was partly grounded in the hope that unnecessary travel and associated costs could be reduced. However, the data do not support presenting AI as a direct solution to poverty or financial hardship. Rather, AI was viewed as potentially useful if it could reduce avoidable travel, improve triage, and help families determine when in‐person specialist care was truly necessary.

### 3.2 Theme 2: AI as a Bridge to Human‐Centred Care

The second major theme describes how participants imagined AI as a possible support mechanism within a constrained rural healthcare system. Participants' expectations were generally hopeful, but their optimism was not unconditional. AI was not described as a replacement for clinicians, Behvarzan, or family caregiving. Rather, participants framed AI as potentially useful when it could extend access to timely advice, support earlier recognition of deterioration, reduce unnecessary travel, and strengthen communication between families and providers.

This theme was closely linked to the structural barriers described in Theme 1. Parents' interest in AI was shaped by their experiences of delayed specialist access, emotional strain, and financial pressure. Providers' interest was shaped by workload, limited specialist availability, and the need for better triage and follow‐up systems. Across interviews, participants' hopes for AI were therefore practical and relational: they valued AI most when it appeared to support human care rather than replace it.

During confirmation sampling, no new major AI‐related use cases emerged. Later interviews continued to emphasise three recurring applications: predictive alerts, emergency guidance, and AI‐supported teleconsultation. This supported the stability of the theme and suggested that participants' expectations were organised around specific care gaps rather than generalised enthusiasm for technology.

#### 3.2.1 Predictive Analytics for Proactive Care

Predictive analytics was one of the clearest ways participants imagined AI contributing to chronic disease management. Parents described the value of early warning tools in relation to uncertainty and fear of delayed response. For them, prediction was not understood as abstract technological innovation; it was meaningful because it could help them recognise deterioration earlier and decide whether urgent care was needed.

One mother described how an early warning system could reduce both clinical uncertainty and the burden of unnecessary travel:

If an app could warn me early that my child's breathing might worsen before I even notice, I wouldn't have to risk long, exhausting trips to the city hospital. For us, this kind of early alert is not just convenient, it's peace of mind in an often‐lonely struggle.

(Mother, P‐10)

This quotation shows that parents associated predictive alerts with reassurance and decision support. The mother did not describe AI as making final clinical decisions. Instead, she imagined AI as a tool that could help her recognise risk earlier and reduce the feeling of being alone when judging whether symptoms were serious. The phrase “peace of mind” is analytically important because it shows that the perceived value of prediction was emotional as well as practical.

Providers also described predictive tools as potentially useful, but their emphasis differed from that of parents. Whereas parents focused on anxiety, travel, and uncertainty at home, providers focused on earlier intervention and improved prioritisation in settings where specialist access was limited:

Predictive AI tools give us a new lens to foresee complications in children without them needing to travel miles. This shifts care from reactive to proactive. In rural areas where specialist visits are scarce, such technology could be the breakthrough we've long awaited.

(General Practitioner, HP‐03)

The provider's account suggests that predictive analytics was valued as a possible triage and monitoring aid. However, the data do not support the conclusion that predictive tools would independently transform rural care. Rather, participants' accounts indicate that such tools would be acceptable only if they were integrated into existing care relationships and linked to clear pathways for human follow‐up.

Across parent and provider interviews, predictive analytics was therefore interpreted as a means of supporting earlier recognition and response, not as autonomous diagnosis. Parents emphasised reassurance, reduced uncertainty, and avoidance of unnecessary travel. Providers emphasised proactive monitoring, prioritisation, and more efficient use of limited specialist resources. This convergence supported the subtheme, while the difference in emphasis showed how the same AI function carried different meanings for families and clinicians.

Evidence of saturation for this subtheme was observed in the final phase of interviews. Later participants continued to describe early alerts, symptom monitoring, and proactive identification of deterioration, but these accounts did not introduce new categories. Instead, they confirmed that prediction was consistently valued when framed as decision support connected to human care. This finding suggests that AI implementation in this context should prioritise transparent risk alerts and clear escalation pathways rather than autonomous clinical decision‐making.

#### 3.2.2 Conversational AI for Emergency Guidance

Conversational AI, including chatbots or virtual assistants, was discussed as a possible source of immediate guidance during acute episodes, particularly when professional help was not readily available. Participants' interest in this use case was closely connected to the timing of emergencies in rural settings. Parents described moments of symptom worsening as situations in which they had to make rapid decisions before reaching a clinic or speaking with a clinician. In this context, conversational AI was valued less as a replacement for emergency care and more as a potential tool for step‐by‐step support while families sought human assistance.

One mother described this need for immediate guidance during a respiratory episode:

In moments when my child suddenly struggles to breathe, having a chatbot that can quickly guide me step‐by‐step until I reach the clinic would be a lifeline. It would be like carrying the doctor's calm voice in my pocket, especially when no one else is around.

(Mother, P‐14)

This account shows that the perceived value of a chatbot was linked to the gap between symptom onset and access to professional care. The mother did not describe the chatbot as independently managing the emergency. Rather, she imagined it as a temporary source of guidance “until I reach the clinic.” The metaphor of “the doctor's calm voice” suggests that parents valued not only information, but also reassurance, sequencing of actions, and reduction of panic during moments of uncertainty.

Providers also saw potential value in conversational AI, but they framed it more in terms of structured guidance and appropriate care‐seeking. One nurse explained:

AI chatbots can democratize emergency care by empowering parents with immediate, personalized advice tailored to their child's situation. This would reduce panic‐induced hospital visits and help families effectively manage emergencies even before professional help arrives.

(Nurse, H‐07)

Although the provider used broad language about “democratizing emergency care,” our interpretation is more cautious. The quotation indicates that providers hoped conversational AI might help parents distinguish between situations requiring urgent referral and situations that could be managed temporarily while seeking advice. However, participants' accounts do not support the conclusion that chatbots could replace emergency services or clinical assessment. Instead, they suggest that conversational AI would need clear safety limits, escalation instructions, and integration with local care pathways.

Across interviews, conversational AI was most acceptable when framed as a supportive tool for urgent guidance rather than as an autonomous emergency decision‐maker. Parents emphasised reassurance, step‐by‐step instructions, and help during moments when they felt alone. Providers emphasised triage support, reduction of panic, and the possibility of guiding families toward appropriate next steps. This difference in emphasis is important: parents focused on emotional and practical support during crisis, whereas providers focused on clinical appropriateness and service use.

Evidence of saturation for this subtheme was seen during later interviews, where participants continued to describe emergency guidance, symptom‐related advice, and stepwise instructions, but did not introduce new categories. The final interviews reinforced that acceptability depended on human oversight and clear referral pathways. Thus, conversational AI was interpreted as potentially useful only when it supported timely connection to care, rather than substituting for professional emergency assessment.

#### 3.2.3 Teleconsultation for Specialist Access

AI‐supported teleconsultation was described as a practical way to reduce the burden of specialist access for families living far from urban pediatric services. This subtheme was closely linked to the geographic and financial barriers described earlier. Parents explained that specialist visits often required long travel, time away from work and household responsibilities, transportation expenses, and uncertainty about whether the visit would result in meaningful clinical guidance. In this context, teleconsultation was valued because it could potentially reduce unnecessary travel while still preserving contact with human specialists.

One parent described the difference between in‐person specialist visits and remote specialist advice:

Before teleconsultation, every visit to the city hospital meant losing an entire day and worrying about my child's condition getting worse. Now, with AI‐assisted virtual visits, I receive expert advice without the need for arduous travel and only take my child to a specialist when necessary.

(Mother, P‐07)

This account shows that teleconsultation was valued not only for convenience, but also for reducing the uncertainty and strain associated with deciding whether a long journey was needed. The parent's emphasis on receiving “expert advice” indicates that acceptability depended on continued access to human clinical judgement. AI was not described as replacing the specialist; rather, it was imagined as supporting the process of determining when specialist review was necessary.

Providers framed AI‐supported teleconsultation in terms of triage, prioritisation, and more efficient use of limited specialist resources. One community health nurse explained:

AI‐powered teleconsultations have transformed the way we manage our caseload. They enable us to prioritize effectively, reduce unnecessary referrals and the burden of travel on families, while ensuring timely specialist access in case of complex cases.

(Community Health Nurse, HCP‐03)

Although this quotation uses strong language about transformation, our interpretation is more measured. The provider's account suggests that AI‐supported teleconsultation was perceived as useful when it helped organise referrals, identify cases requiring specialist input, and reduce unnecessary travel for families. However, the data do not support treating teleconsultation as a complete solution to specialist shortages. Its value depended on whether virtual consultations were linked to reliable referral pathways, available clinicians, adequate digital infrastructure, and clear responsibility for follow‐up.

Across interviews, teleconsultation was the AI‐related application most directly connected to participants' existing experiences of access barriers. Parents emphasised reduced travel, lower indirect costs, and quicker reassurance from specialists. Providers emphasised triage, caseload management, and more appropriate use of scarce specialist time. These perspectives converged around the idea that teleconsultation could extend access to human expertise, but not replace the need for in‐person care when physical examination, urgent intervention, or complex management decisions were required.

Evidence of saturation for this subtheme was observed in the later interviews. Participants continued to describe virtual specialist access, referral prioritisation, travel reduction, and timely advice, but no new categories emerged during confirmation sampling. Later accounts mainly clarified the conditions under which teleconsultation would be acceptable: reliable internet connection, involvement of trusted rural providers or Behvarzan, clear escalation routes, and reassurance that complex cases would still be referred for in‐person specialist care.

Thus, AI‐supported teleconsultation was interpreted as a potentially feasible bridge between rural families and urban specialists. Its perceived value lay in reducing avoidable travel and improving triage, while maintaining human clinical oversight and preserving access to in‐person specialist care when needed.

### 3.3 Theme 3: Trust Dynamics and Cultural Considerations

The third major theme describes the conditions under which participants considered AI acceptable or unacceptable in rural pediatric chronic care. Across interviews, trust did not depend only on whether AI was perceived as useful. Participants also evaluated AI in relation to human oversight, privacy, professional responsibility, cultural expectations, and the quality of relationships between families and healthcare providers.

Parents and providers were not uniformly resistant to AI. Rather, they expressed conditional trust. AI was acceptable when it supported human decision‐making, improved access to advice, and reduced practical burdens. It became concerning when participants imagined it replacing clinical judgement, weakening relationships with providers, or making decisions without accountability. This theme therefore helps explain why participants could simultaneously express hope about AI‐supported care and skepticism about autonomous AI decision‐making.

During confirmation sampling, later interviews did not introduce new trust‐related categories. Instead, they reinforced three recurring conditions for AI acceptability: human oversight, protection of sensitive health data, and preservation of human connection in care.

#### 3.3.1 Parental Trust in Human Oversight

Human oversight was central to parents' trust in AI. Parents described AI as potentially useful for reminders, alerts, education, or guidance, but they were hesitant to trust AI with decisions they perceived as serious, uncertain, or life‐affecting. Their concerns were not simply about lack of familiarity with technology. Rather, parents distinguished between technical support and moral‐clinical responsibility. AI could help organise information, but a clinician was still expected to interpret the child's situation, consider context, and take responsibility for decisions.

One mother explained this distinction clearly:

I want to use technology to help my child, but I cannot trust a machine to make decisions that could affect their life. AI might be able to remind me of medications or alert me to symptoms, but when it comes to serious choices, I need a real doctor who can see and understand my child's unique needs.

(Mother, P‐09)

This quotation shows that the mother's concern was not a general rejection of AI. She accepted AI for lower‐risk supportive functions, such as medication reminders or symptom alerts, but rejected autonomous decision‐making in serious situations. The phrase “see and understand my child's unique needs” indicates that trust was linked to individualized clinical judgement and relational recognition. For parents, a trustworthy care system was one in which AI could support vigilance but not replace the clinician's responsibility.

Providers described similar concerns when reflecting on parents' reactions to AI. One community health nurse stated:

While AI holds the promise of supporting families, I have seen parents hesitant because they fear it will replace human judgment. Our challenge is to help them see AI as a tool, not a decision‐maker, and to ensure that human oversight remains central to care decisions.

(Community Health Nurse, H‐12)

The provider's account supports the interpretation that trust depends on how AI is introduced and explained. Parents' hesitation was not interpreted by providers as irrational resistance; rather, it was seen as a response to uncertainty about whether AI would supplement or displace human judgement. This suggests that implementation would require clear communication about the boundaries of AI, the role of clinicians, and the process by which AI‐generated recommendations would be reviewed.

Across the dataset, parents consistently drew a boundary between supportive AI and autonomous AI. Supportive AI was associated with reminders, alerts, symptom guidance, and easier access to advice. Autonomous AI was associated with risk, uncertainty, and loss of human accountability. Providers similarly emphasised the need to frame AI as a decision‐support tool rather than a decision‐maker. This convergence across participant groups strengthened the interpretation of human oversight as a central condition of trust.

Evidence of saturation for this subtheme was observed during the final interviews. Later participants continued to express openness to AI‐supported reminders, alerts, and guidance while resisting the idea of AI making serious clinical decisions independently. These later interviews did not generate new trust categories; instead, they confirmed the stability of human oversight as a core requirement for AI acceptability in rural pediatric chronic care.

Thus, parental trust in AI was conditional rather than absent. Participants did not reject AI because it was technological; they questioned whether it would preserve human responsibility, individualized judgement, and relational care. These findings suggest that AI systems in this context should be designed and presented as transparent decision**‐**support tools with clear mechanisms for clinician review, escalation, and accountability.

#### 3.3.2 Provider Concerns About Data, Workload, and Professional Accountability

Concerns about AI were not limited to parents. Healthcare providers also raised questions about how AI would affect data privacy, workload, professional accountability, and clinical relationships. Providers did not reject AI as a category; rather, they questioned whether rural health systems had the governance structures, training resources, and staffing capacity needed to use AI safely. Their concerns therefore reflected both ethical issues and practical implementation challenges.

Although this subtheme focuses primarily on provider concerns, parents also linked data security to relational trust. One mother explained:

I want my child's information to be secure, but I also worry that if doctors rely too much on AI, they might lose their personal connection. Sometimes, I feel like machines don't understand the fears and hopes we have with ourselves trust isn't just about data; it's about feeling heard.

(Mother, P‐15)

This quotation shows that privacy was not understood only as technical data protection. For parents, trust also involved feeling recognised by healthcare professionals and not reduced to data points or algorithmic outputs. The phrase “trust isn't just about data; it's about feeling heard” indicates that privacy, communication, and relational care were intertwined. This parent perspective helps contextualise why providers' later concerns about explaining AI decisions were important: families expected both protection of information and continued human engagement.

Providers described these concerns from the perspective of responsibility and workload. One nurse stated:

AI can be powerful, but if we don't have strong rules about data privacy or training on how to use it, we're not only violating patients' trust, but we're also putting a huge burden on staff who have to constantly monitor these tools and explain every AI decision to worried families.

(Nurse, HP‐07)

This account highlights two linked provider concerns. First, AI implementation was viewed as requiring clear rules for data privacy and patient protection. Second, providers anticipated that AI might increase rather than reduce workload if staff were expected to monitor outputs, interpret recommendations, and explain algorithmic decisions without adequate training or institutional support. Thus, provider concern was not simply resistance to technology or fear of losing authority. It reflected uncertainty about who would be accountable for AI‐supported recommendations and how additional explanatory work would be absorbed in already constrained rural clinics.

Across provider interviews, concerns about AI clustered around three recurring issues: protection of patient data, adequacy of training, and preservation of professional responsibility. Providers were particularly concerned that poorly implemented AI could create new burdens by requiring them to verify AI outputs, reassure families, and mediate disagreements between algorithmic recommendations and clinical judgement. Parents, in turn, reinforced the importance of these concerns by emphasising that data security alone would not be enough if AI weakened communication with clinicians.

Evidence of saturation for this subtheme was observed during the final interviews. Later provider accounts continued to return to privacy, training, workload, and accountability, but did not introduce new categories. Instead, they clarified that acceptability depended on practical safeguards: clear data governance, defined responsibility for AI‐supported decisions, training for staff, and transparent communication with families.

These findings suggest that provider concerns should be interpreted as implementation conditions rather than simple resistance. AI‐supported care would need to include explainable outputs, clear escalation procedures, staff training, and institutional policies that protect patient data while preserving professional accountability.

#### 3.3.3 Cultural Values and Human Connection

Human connection was a recurring condition of trust in participants' accounts. Parents and providers emphasised that some aspects of chronic illness care, particularly discussions involving fear, uncertainty, deterioration, or major treatment decisions, required direct human presence and emotional responsiveness. Participants did not describe this preference as opposition to technology. Rather, they drew a boundary between information or guidance that AI might provide and the relational reassurance they associated with trusted healthcare professionals.

One father explained this distinction:

No machine can replace the comfort I get when my doctor looks me in the eye and understands my fears, not just my symptoms. AI advice might tell me what medication to give, but only a human can hold my hand and truly care.

(Father, P‐12)

This quotation shows that trust was not based only on receiving correct medical information. The father valued being seen, understood, and emotionally supported by a clinician. His distinction between “symptoms” and “fears” is analytically important because it indicates that participants understood care as both clinical and relational. AI could potentially provide advice, but participants did not believe it could fully respond to the emotional and contextual dimensions of caregiving.

Providers expressed a similar view, particularly when discussing sensitive conversations with families. One community health worker stated:

In these families, recovery is about more than just treatment; it's about the trust that builds face‐to‐face. Technology can help, but it can't replicate the empathy and reassurance that only a human caregiver can provide during sensitive discussions.

(Community Health Worker, H‐07)

This provider account reinforces the interpretation that acceptability of AI depended on whether it preserved, rather than weakened, therapeutic relationships. Providers did not reject technology, but they emphasised that face‐to‐face trust remained important when families needed reassurance, explanation, or emotional support. The phrase “technology can help” is important because it shows conditional openness: AI was acceptable when it supported care processes, but not when it replaced human presence in relationally sensitive moments.

Across interviews, participants repeatedly distinguished between informational support and relational care. Parents emphasised comfort, being heard, and confidence that someone understood their child's situation. Providers emphasised empathy, reassurance, and the importance of maintaining trust during sensitive discussions. These accounts suggest that human connection functioned as a boundary condition for AI acceptability: participants were willing to consider AI for reminders, alerts, triage, or access support, but they expected emotionally significant decisions and conversations to remain human‐led.

Evidence of saturation for this subtheme was observed during the confirmation phase of data collection. Later interviews continued to reinforce the same distinction between AI as a source of information and healthcare professionals as sources of empathy, reassurance, and accountable judgement. No new categories emerged in relation to cultural values or human connection; instead, later accounts confirmed that preservation of relational care was a stable requirement across parent and provider perspectives.

These findings suggest that AI implementation in this context should be framed as supporting human connection rather than replacing it. Participants' accounts indicate that AI may be more acceptable when it reduces administrative or access‐related burdens and enables providers to focus on communication, explanation, and emotionally responsive care.

### 3.4 Implementation Pathways

Participants did not discuss AI only in terms of possible benefits or risks; they also described conditions that would make AI more acceptable and usable in rural pediatric chronic care. These implementation pathways emerged from participants' reflections on the barriers identified in earlier themes. Across interviews, three conditions were repeatedly emphasised: digital literacy and hands‐on training, integration with the existing Behvarz network, and co‐design of AI tools with attention to local cultural beliefs and caregiving practices.

These pathways should not be interpreted as formal implementation recommendations generated independently by the researchers. Rather, they reflect participants' own accounts of what would be necessary for AI‐supported care to fit everyday rural healthcare realities. During confirmation sampling, no new implementation pathways emerged; later interviews reinforced the same three conditions and provided additional examples of why training, trusted intermediaries, and cultural adaptation would be necessary.

#### 3.4.1 Digital Literacy and Training

Digital literacy was identified as a practical condition for AI acceptability, particularly among caregivers with limited experience using health‐related applications. Parents' concerns were not limited to whether they could operate a device. Some worried that misunderstanding an app, pressing the wrong button, or misinterpreting AI‐generated advice could negatively affect their child's care. For these participants, training was important because it could reduce fear, increase confidence, and clarify the limits of what AI tools could and could not do.

One mother described how guided instruction changed her perception of the technology:

As a mother in the countryside, I was more worried about using new apps sometimes the buttons confuse me, and I think, ‘What if I press the wrong button and hurt my child?’ But when the health worker showed me step by step, I felt like this technology had become a friend to help me, not a stranger to fear.

(Mother, P‐12)

This account shows that reluctance to use AI was not necessarily resistance to innovation. Rather, fear emerged from uncertainty about how to use the tool safely. The mother's shift from describing technology as “a stranger to fear” to “a friend to help me” suggests that trust was built through interpersonal guidance, not through the technology alone. The role of the health worker was therefore central: the tool became acceptable when introduced by a trusted person who could explain it in familiar terms.

Providers similarly emphasised that training would need to be continuous, practical, and adapted to users' abilities. One rural nurse practitioner explained:

“In my experience, if we don't patiently guide parents through each feature, many elderly caregivers simply stop using AI apps. Training is not optional; it's the bridge that turns these digital tools from sophisticated machines into reliable assistants in daily care.”

*(Rural Nurse Practitioner, H‐07)*

The provider's account reinforces that training was viewed as an implementation requirement rather than a one‐time technical orientation. The phrase “patiently guide parents through each feature” suggests that effective training would need to be gradual, hands‐on, and responsive to caregiver confidence. It also indicates that usability problems could lead to abandonment of AI tools, especially among older caregivers or those with limited digital experience.

Across interviews, parents emphasised fear of error, confusion, and the need for trusted step‐by‐step explanation. Providers emphasised the risk of non‐use or misuse if AI tools were introduced without adequate training. These perspectives converged around the idea that digital literacy was not only an individual skill but also a relational and system‐level responsibility. AI tools would need to be accompanied by accessible instruction, opportunities for repeated practice, and ongoing support from trusted rural health workers.

Evidence of saturation for this subtheme was observed during later interviews. Participants continued to mention difficulty using apps, fear of mistakes, need for demonstration, and dependence on trusted health workers for explanation, but no new categories emerged. Later interviews mainly reinforced the importance of training as a precondition for safe and sustained AI use.

These findings suggest that AI implementation in rural pediatric chronic care should include structured digital literacy support for both caregivers and providers. Training should address not only how to operate AI tools, but also how to interpret alerts, when to seek human advice, how to protect personal health information, and what limits should be placed on AI‐generated recommendations.

#### 3.4.2 Integration With the Behvarz Network

Integration with the existing Behvarz network emerged as a central condition for acceptable AI implementation. Participants did not describe AI as a standalone system that families would use independently or as a replacement for rural healthcare workers. Instead, they emphasised that AI would be more trusted and useful if it were introduced through familiar community health workers who already had relationships with families and knowledge of local care pathways.

Parents often described Behvarzan as the first point of contact when they were uncertain about symptoms, referrals, or the need to travel to urban centres. In this context, AI was imagined as a tool that could strengthen the Behvarz role by supporting triage and referral decisions. One mother explained:

In our village, health workers are lifesavers, but sometimes they struggle to know who really needs to go to the city for care. If an AI tool can quietly help them make decisions faster and more accurately, it will mean fewer unnecessary trips and more timely help for my child. It's like they've been given an intelligent assistant that never sleeps.

(Mother, P‐12)

This account shows that the mother's trust was directed primarily toward the health worker, not the AI tool alone. AI was acceptable because it was imagined as supporting someone already embedded in the community. The phrase “an intelligent assistant that never sleeps” suggests that parents valued the possibility of continuous support, but the support was still expected to operate through human care relationships. The mother did not describe AI as replacing Behvarzan; rather, she saw it as helping them make better and faster decisions.

Providers similarly viewed AI as potentially useful when it could assist, rather than displace, clinical judgement. One community health worker stated:

Putting AI into our health facilities will transform the way we assess children with complex conditions. This is not about replacing our judgment it's about augmenting it AI can pick up on subtle signs that we might miss when we're overwhelmed with decision‐making, ensuring that urgent cases aren't missed. This support empowers us to act with confidence and reduce delays in referrals.

(Community Health Worker, HCP‐07)

Although the provider used strong language about transformation, the analytic meaning of this quotation is more specific. The provider valued AI as a possible aid for recognising risk, prioritising referrals, and reducing missed warning signs when workload was high. This suggests that AI was understood as a decision‐support tool within the Behvarz system, not as an independent authority. The provider's reference to being “overwhelmed with decision‐making” also links this subtheme to earlier findings on rural workforce constraints and limited specialist access.

Across interviews, parents and providers converged around the idea that AI should be embedded in existing rural care relationships. Parents emphasised trust in Behvarzan, reduced unnecessary travel, and timely help for children. Providers emphasised support for triage, confidence in referral decisions, and reduction of missed urgent cases. These accounts suggest that the Behvarz network could function as an important human interface between families and AI‐supported tools.

Evidence of saturation for this subtheme was observed during confirmation sampling. Later interviews continued to describe Behvarzan as trusted intermediaries, AI as a support for triage, and referral decisions as a key point where AI could be useful. No new categories emerged in relation to implementation through the Behvarz network; instead, later accounts reinforced the importance of embedding AI within existing rural healthcare structures rather than introducing it as a separate digital system.

These findings suggest that AI implementation in rural pediatric chronic care may be more acceptable if it strengthens the existing Behvarz network. Such implementation would require training Behvarzan to use AI tools, defining clear referral protocols, ensuring clinician oversight, and communicating to families that AI is intended to support not replace the trusted community health workers who already mediate access to care.

#### 3.4.3 Co‐Design and Cultural Resonance

Co‐design with families and rural healthcare providers emerged as another condition for acceptable AI implementation. Participants emphasised that AI tools would need to reflect the everyday realities, language, beliefs, and care practices of rural families. Their concern was not simply whether AI tools would be technically accurate, but whether they would be understandable, trustworthy, and compatible with local ways of making sense of illness and care.

One mother explained this need for cultural fit by referring to familiar health beliefs and household practices:

For technology to really help us, it needs to understand our world not just medicine, but our customs, like how we believe in ‘hot’ and ‘cold’ foods for healing. If AI ignores this, it's like an outsider telling us what to do, not a companion to accompany us.

(Mother, P‐12)

This quotation shows that cultural resonance was linked to trust and perceived relevance. The mother was not asking AI to replace biomedical guidance with traditional beliefs. Rather, she was expressing concern that a tool designed without knowledge of local practices might feel alien, dismissive, or difficult to integrate into everyday caregiving. The phrase “an outsider telling us what to do” suggests that acceptability depends partly on whether families feel respected and understood by the technology and by those implementing it.

Providers also emphasised the importance of involving families in the design process. One community health nurse stated:

We found that AI tools designed without input from families ignore the critical cultural beliefs that guide health behaviors here. Co‐creating these platforms with parents, while respecting their traditional perspectives and practices, is the only way we can make AI accepted and effective in rural care.

(Community Health Nurse, H‐07)

The provider's account supports the interpretation that cultural adaptation was viewed as an implementation requirement rather than a superficial translation exercise. However, the data should not be interpreted as suggesting that all local beliefs should be incorporated uncritically into clinical recommendations. Rather, participants' accounts indicate that AI tools would need to acknowledge local beliefs, communicate respectfully, and create space for healthcare providers to explain where traditional practices align or conflict with safe chronic disease management.

Across interviews, parents emphasised familiarity, respect, understandable language, and recognition of household practices. Providers emphasised the importance of family input, culturally appropriate communication, and avoiding top‐down implementation. These perspectives converged around the idea that co‐design could help prevent AI tools from being perceived as externally imposed or disconnected from rural caregiving realities.

Evidence of saturation for this subtheme was observed during the confirmation phase of interviews. Later participants continued to describe the need for culturally familiar language, family involvement, respect for local beliefs, and adaptation to rural caregiving routines, but no new categories emerged. Instead, later interviews reinforced co‐design and cultural fit as stable conditions for AI acceptability.

Taken together, the findings reveal that participants' expectations of AI were shaped by both structural need and relational caution. Parents and providers identified substantial barriers in rural pediatric chronic care, including specialist distance, caregiver burden, financial strain, limited referral support, and workforce pressure. They also imagined specific AI‐supported applications, including predictive alerts, emergency guidance, and teleconsultation. However, acceptability depended on clear conditions: AI should support rather than replace human judgement, protect privacy, preserve relational care, be introduced through trusted rural health workers, and be designed with attention to local language, beliefs, and caregiving practices.

The central finding is therefore not that participants were simply optimistic or skeptical about AI. Rather, they expressed conditional openness: AI was viewed as potentially valuable when it extended access to human‐centred care, but problematic when imagined as autonomous, culturally detached, or disconnected from existing rural healthcare relationships.

## 4 Discussion

This qualitative study explored parent and healthcare provider expectations of AI as a possible bridge to human‐centred care for children with chronic illness in rural Iran. The findings show that participants' expectations were shaped by both structural need and relational caution. Three major themes were identified: structural barriers to chronic care, including geographic access challenges, caregiver burden, and financial strain; AI as a potential bridge to human‐centred care, including predictive analytics, conversational AI, and teleconsultation; and trust dynamics and cultural considerations that shaped participants' conditional acceptance of AI.

Rather than expressing simple optimism or resistance, participants described AI as potentially useful when it supported timely access to human expertise, reduced avoidable travel, and strengthened decision‐making within existing care relationships. However, they were cautious about AI applications that might replace clinician judgement, weaken trust, increase workload, or fail to reflect local caregiving practices.

### 4.1 Interpretation of Findings in Relation to Existing Literature

The findings contribute to the growing literature on AI adoption in healthcare by showing how expectations of AI are shaped by the everyday realities of rural chronic care. Participants' accounts of geographic isolation, workforce pressure, caregiver burden, and financial strain are consistent with previous research documenting access barriers in rural healthcare and primary care workforce limitations in resource‐constrained settings [29, 30]. These structural barriers are important because they shaped the specific forms of AI that participants considered useful. AI was not valued as an abstract innovation, but as a possible response to practical problems such as delayed specialist access, uncertainty during symptom worsening, travel costs, and limited referral support.

#### 4.1.1 Theme 1: Structural Barriers to Chronic Care

The first theme highlights the systemic challenges that shaped chronic illness care for children in rural Iran. Geographic and specialist access barriers were central to both parent and provider accounts. Parents described specialist care as physically distant, time‐consuming, and emotionally stressful, while providers described the difficulty of managing complex pediatric cases with limited specialist backup. This finding is consistent with rural healthcare literature showing that distance, transport difficulties, and limited local services remain major barriers to timely care [29, 30].

Caregiver burden and emotional isolation also emerged as important dimensions of structural vulnerability. Mothers in particular described caregiving as meaningful but exhausting, with chronic illness management becoming a continuous responsibility that shaped daily routines, emotional well‐being, and family life. This finding is consistent with international literature showing that parents of children with chronic illness or disability often experience high levels of stress, emotional burden, and social isolation. However, the present study adds contextual detail by showing how caregiving burden in rural Iran was shaped by expectations of maternal responsibility, limited access to specialist support, and the absence of routine psychosocial support for caregivers. Financial strain was another major barrier. Parents described the costs of transportation, medication, missed work, and repeated specialist visits as cumulative pressures that affected household resources. Providers similarly reported that financial hardship contributed to delayed follow‐up, missed appointments, and worsening clinical presentations. This finding is consistent with literature on out‐of‐pocket health spending and financial risk in low‐ and middle‐income countries, where healthcare costs can reduce families' ability to meet daily needs and can deepen existing vulnerability [31, 32, 33]. In the present study, financial strain was not separate from geographic access barriers; rather, distance from specialist care made each episode of care more expensive and disruptive.

Providers' accounts also showed that rural care constraints were experienced at the service level. Workforce shortages, referral limitations, administrative burden, and limited infrastructure affected providers' ability to deliver timely and continuous care. These findings are consistent with evidence that primary care workforce development in resource‐limited settings is constrained by workload, training needs, limited support systems, and uneven infrastructure [30]. Together, these findings suggest that AI implementation should not be discussed separately from the structural conditions of rural care. AI tools are more likely to be meaningful when they address specific access and workflow problems rather than when they are introduced as standalone technologies.

#### 4.1.2 Theme 2: AI as a Bridge to Human‐Centred Care

The second theme shows that participants imagined AI as potentially useful when it could reduce delays, support earlier recognition of deterioration, improve access to advice, and help families and providers make better decisions. This finding is consistent with broader literature suggesting that AI may support healthcare through prediction, risk stratification, decision support, remote monitoring, and workflow improvement, while also requiring careful attention to safety, validation, and clinical integration [16, 34, 35, 36, 37].

Predictive analytics was one of the clearest AI applications described by participants. Parents valued early alerts because they could reduce uncertainty and provide reassurance during home‐based caregiving. Providers valued predictive tools because they could support triage, prioritisation, and earlier intervention in settings where specialist access was limited. This finding is consistent with literature suggesting that AI‐enabled monitoring and predictive tools may support earlier identification of clinical deterioration, although their safe use depends on validation, explainability, human oversight, and integration into clinical workflows [17, 36, 37]. Importantly, participants did not describe predictive AI as autonomous diagnosis. Rather, they valued it as decision support that could help families and providers recognise when human care was needed.

Conversational AI for emergency guidance was also viewed as potentially useful, especially during the interval between symptom worsening and access to professional care. Parents' desire for “the doctor's calm voice” in moments of crisis suggests that conversational tools were valued not only for information, but also for reassurance and step‐by‐step guidance. This is consistent with literature on patient‐facing conversational and voice‐assistant technologies, which suggests that such tools may support self‐management, information access, and behavioural guidance, while also raising concerns about safety, personalisation, and escalation to human care [34]. In the present study, participants' accounts indicate that emergency guidance tools would need clear safety boundaries, explicit referral instructions, and integration with local care pathways.

Teleconsultation for specialist access was another concrete and practical AI‐supported application. Parents valued the possibility of receiving expert advice without repeated travel to urban hospitals, while providers viewed AI‐supported teleconsultation as a possible way to improve referral prioritisation and make better use of limited specialist resources. This finding is consistent with telemedicine literature suggesting that remote consultation may reduce geographic barriers in rural settings, although its effectiveness depends on infrastructure, affordability, digital literacy, and integration with existing services [35]. In this study, teleconsultation was not viewed as a replacement for specialist care. Instead, it was valued as a way to extend access to human expertise while preserving pathways for in‐person assessment when needed.

Overall, Theme 2 suggests that participants' optimism about AI was highly practical. They were not primarily interested in AI as a futuristic technology, but in tools that could reduce avoidable travel, provide timely advice, support triage, and connect rural families with human clinicians more effectively. This supports the central interpretation of AI as a potential bridge to human‐centred care rather than as a substitute for care.

#### 4.1.3 Theme 3: Trust Dynamics and Cultural Considerations

The third theme highlights the trust conditions that shaped participants' acceptance of AI. Participants did not evaluate AI only in relation to perceived usefulness or technical performance. Rather, they considered whether AI would preserve human oversight, protect sensitive information, maintain relational care, and fit local cultural expectations. This finding is consistent with broader literature showing that trust in AI depends on perceived competence, transparency, accountability, and alignment with human values [8, 36, 37, 38, 39].

Parental trust in human oversight emerged as a central condition for AI acceptability. Parents expressed openness to AI‐supported reminders, alerts, and preliminary guidance, but they were reluctant to accept autonomous AI decision‐making in serious clinical situations. Their concerns were not simply about unfamiliarity with technology; rather, they reflected a distinction between technical support and accountable clinical judgement. The statement “I need a real doctor who can see and understand my child's unique needs” illustrates that trust was relational as well as informational. This finding is consistent with studies showing that patients and the public may be receptive to clinical AI when it supports, rather than replaces, human professionals [8]. It also aligns with work on explainability and human oversight in healthcare AI, which emphasises that AI systems must remain understandable, reviewable, and clinically accountable [17, 37].

Provider concerns about data, workload, and professional accountability further demonstrate that AI implementation cannot be reduced to technical adoption. Providers raised concerns about data privacy, staff training, responsibility for AI‐supported recommendations, and the additional labour of explaining AI outputs to families. These concerns are consistent with literature identifying governance, explainability, workflow integration, and clinician interaction with AI‐generated recommendations as central challenges for safe AI implementation in healthcare [17, 37, 39]. In the present study, provider hesitation should therefore be interpreted less as resistance to innovation and more as concern about whether rural health systems have the infrastructure, training, and governance needed to use AI responsibly.

Cultural values and human connection also shaped participants' expectations. Parents and providers emphasised that sensitive conversations, serious clinical decisions, and moments of fear required empathy, reassurance, and human presence. The distinction between “AI advice” and “a human who can hold my hand” indicates that participants viewed care as fundamentally relational. This finding extends general discussions of trust in AI by showing that, in this rural care context, acceptability depended not only on technical reliability but also on whether AI preserved the human connection that families associated with trustworthy care [8, 38]. Therefore, AI should be positioned as a tool that supports communication, triage, and access, rather than as a replacement for emotionally responsive clinical relationships.

#### 4.1.4 Implementation Pathways

Participants identified several conditions that would make AI implementation more acceptable and feasible in rural pediatric chronic care. These included digital literacy support, integration with the Behvarz network, and co‐design with families and rural providers. These implementation pathways show that participants' expectations were not limited to whether AI could work technically; they were also concerned with how AI would be introduced, explained, governed, and embedded in existing care relationships.

Digital literacy emerged as an important implementation requirement, particularly for older caregivers and those with limited experience using health applications. Parents described fear of making mistakes, misunderstanding app functions, or misinterpreting AI‐generated advice. Providers similarly emphasised that training would be necessary to prevent non‐use, misuse, or abandonment of AI tools. This finding is consistent with literature showing that digital literacy training for marginalised or vulnerable populations must address usability, confidence, support, and contextual barriers rather than focusing only on technical instruction [17]. In this study, digital literacy was not only an individual skill; it was a relational process that depended on patient guidance from trusted health workers.

Integration with the Behvarz network emerged as a particularly important implementation pathway. Participants did not describe AI as a standalone system for families to use independently. Instead, they imagined AI as a support tool for trusted rural health workers who already mediate access between families and the formal healthcare system. This finding is consistent with socio‐technical approaches to health information technology, which emphasise that digital tools are more likely to be accepted when they fit existing workflows, roles, relationships, and organisational structures [40, 41, 42]. Embedding AI within the Behvarz network may therefore be more acceptable than introducing AI as a separate digital intervention because it preserves the trusted human interface between rural families and the healthcare system.

Co‐design and cultural resonance were also emphasised as essential. Participants stressed that AI platforms should be developed with input from families and rural providers so that they are understandable, respectful, and compatible with local caregiving practices. References to “hot” and “cold” foods illustrate that families evaluated AI not only according to biomedical usefulness, but also according to whether it recognised local beliefs and everyday caregiving routines. This does not mean that AI tools should reproduce traditional beliefs uncritically. Rather, it suggests that culturally resonant design should allow respectful communication, clarification, and dialogue between families and healthcare professionals. This finding is consistent with patient‐centred and socio‐technical approaches to AI implementation, which emphasise that digital health tools must be designed for the social context in which they will be used [17, 37, 42].

### 4.2 Theoretical Context and Framework Integration

Although this study used conventional qualitative content analysis rather than a theory‐driven design, the findings can be interpreted through several theoretical perspectives relevant to healthcare technology adoption. These frameworks help clarify why participants expressed conditional openness toward AI rather than simple acceptance or rejection.

#### 4.2.1 Technology Acceptance Model

The findings partly align with the Technology Acceptance Model, which suggests that perceived usefulness and perceived ease of use influence technology acceptance. Participants perceived AI as potentially useful when it addressed concrete access problems, such as delayed specialist care, uncertainty during acute episodes, and unnecessary travel. At the same time, concerns about digital literacy, reliability, and interpretation of AI‐generated advice indicate that ease of use remains a major condition for adoption in rural settings. However, the findings also suggest that conventional technology acceptance models are insufficient on their own. Participants' expectations were shaped by trust in Behvarzan, cultural preferences for human interaction, privacy concerns, infrastructure limitations, and the perceived seriousness of the clinical situation. For example, parents were more willing to consider AI for reminders or early alerts than for autonomous clinical decision‐making. This suggests that perceived usefulness should be understood by use case, level of risk, and degree of human oversight. This interpretation is consistent with diffusion‐oriented perspectives, which emphasise that adoption depends not only on usefulness but also on compatibility with social systems, complexity, and local context [37, 38, 39, 40, 41, 42, 43].

#### 4.2.2 Trust in Technology Framework

Participants' concerns are also consistent with trust‐in‐technology literature, which emphasises that trust in AI depends on perceived competence, reliability, transparency, benevolence, and accountability [38]. Parents and providers described specific conditions under which they would trust AI: clear human oversight, protection of patient data, transparent recommendations, consistency with clinical judgement, and the ability to escalate to human care when needed.

The present findings add contextual detail by showing that trust was also culturally and relationally shaped. Participants wanted AI to respect local beliefs, preserve human connection, and operate through familiar rural care relationships. In this sense, trust was not only technical or institutional; it was also relational and cultural. This is consistent with Middle Eastern studies showing that AI acceptance in healthcare is shaped by perceived usefulness, trust, privacy, professional readiness, and integration with clinical workflows [43, 44, 45, 46]. However, the present study extends this literature by focusing on rural pediatric chronic care and by including both family caregivers and frontline rural healthcare providers.

#### 4.2.3 Socio‐Technical Systems Perspective

Although the present study used inductive conventional content analysis rather than a theory‐driven analytic framework, the findings can be interpreted through a socio‐technical systems perspective. Socio‐technical systems thinking originated from work showing that organisational performance and worker experience are shaped by the interaction between social arrangements and technical systems, rather than by technology alone. In healthcare, this perspective has been used to understand why health information technologies succeed or fail depending on their fit with people, workflows, organisational routines, communication practices, physical infrastructure, and external policy environments. This perspective is relevant to the present findings because participants did not evaluate AI only in terms of technical capability. Rather, their expectations were shaped by the broader rural care system in which AI would be introduced. Parents emphasised trust, emotional reassurance, human oversight, cultural beliefs, and the continuing role of clinicians and Behvarzan. Providers emphasised workflow burden, data privacy, training needs, referral processes, and the risk that AI could disrupt rather than support clinical relationships. These findings suggest that AI implementation in rural Iran would require alignment between technical functions, such as prediction, triage, and teleconsultation, and the social and organisational realities of rural pediatric chronic care. The emphasis on embedding AI within the Behvarz network further illustrates this socio‐technical interpretation. Participants did not describe AI as a standalone replacement for human care; rather, they imagined it as a tool that could support existing community‐based relationships, strengthen referral decisions, and extend access to specialist advice while preserving human accountability. This finding is consistent with socio‐technical approaches to health information technology, which emphasise that digital tools must be designed and evaluated as part of complex adaptive healthcare systems rather than as isolated technical interventions [40].

### 4.3 Comparison With International Literature

#### 4.3.1 Similarities With High‐Income Country Findings

Participants' conditional optimism about AI is consistent with findings from international literature showing that patients, the public, and healthcare professionals may view AI as useful when it improves access, supports diagnosis, enhances monitoring, or assists clinical decision‐making. However, this optimism is often accompanied by concerns about transparency, accountability, privacy, and the need for continued human oversight [8, 17, 37]. In the present study, parents and providers similarly valued AI when it was framed as a supportive tool, but expressed caution when AI was imagined as replacing human judgement or weakening clinical relationships.

The trust dynamics identified in this study also mirror findings from broader AI acceptance literature. Systematic reviews and conceptual work on trust in AI have shown that acceptance depends not only on perceived usefulness, but also on perceived reliability, explainability, benevolence, and accountability [8, 37, 38]. In our study, these issues were expressed through participants' insistence that AI‐generated recommendations should be understandable, reviewable, and connected to human care. Parents' preference for a “real doctor” in serious decisions and providers' concerns about explaining AI outputs to families both reinforce the importance of human oversight in clinical AI implementation.

#### 4.3.2 Differences and Context‐Specific Factors

Despite these similarities, the present findings also differ from much of the literature from high‐income settings. In many high‐income country studies, AI acceptance is discussed mainly in relation to physician‐patient interaction, hospital‐based decision‐making, or institutional digital readiness. In contrast, participants in this study placed strong emphasis on the Behvarz network as a trusted intermediary between families and the formal healthcare system. This reflects the distinctive structure of rural primary healthcare in Iran and suggests that AI implementation in this context may be more acceptable when introduced through community‐based health workers rather than directly to families as a standalone digital tool.

Infrastructure concerns were also more prominent in the present study than in many high‐income country discussions of AI adoption. Participants described internet connectivity, digital literacy, training, and local support as prerequisites for safe and sustained use. These concerns are consistent with literature showing that digital health and AI implementation in resource‐limited settings depends on infrastructure, workforce capacity, governance, and integration with existing services [17, 30, 35]. Therefore, AI tools developed for highly resourced settings cannot be assumed to transfer directly to rural contexts without adaptation.

The financial strain described by participants also represents an important context‐specific issue. Parents' accounts showed that the costs of travel, medication, lost time, and repeated specialist visits could affect household resources and delay care‐seeking. This finding is consistent with evidence that out‐of‐pocket health spending can create financial risk for families in low‐ and middle‐income countries [31]. In the present study, interest in AI‐supported teleconsultation and triage was partly shaped by the hope that unnecessary travel and related costs could be reduced. However, AI should not be presented as a solution to poverty or financial hardship. Rather, its potential contribution lies in reducing avoidable care‐seeking burdens when safely integrated into referral and follow‐up systems.

Overall, comparison with international literature suggests that some concerns about AI are shared across settings, including trust, explainability, privacy, and human oversight. However, the present study adds context‐specific insight by showing how these concerns are shaped by rural infrastructure, the Behvarz network, caregiver burden, financial strain, and culturally grounded expectations of relational care. This supports the interpretation that AI implementation in rural pediatric chronic care should be locally adapted, human‐centred, and embedded within existing care relationships.

#### 4.3.3 Comparison With Other Middle Eastern Contexts

Empirical research on perceptions of AI in healthcare across Middle Eastern settings remains limited, particularly in relation to pediatric chronic illness, rural populations, and community‐based primary care. Existing regional literature has more commonly examined public perceptions, physician attitudes, and healthcare professionals' readiness for AI in general clinical settings. Studies from Saudi Arabia, the United Arab Emirates, and Bahrain suggest that AI acceptance in healthcare is shaped by perceived usefulness, trust, data privacy, professional readiness, training, and the extent to which AI tools can be integrated into clinical workflows [43, 44, 45, 46].

These concerns are consistent with the present findings, in which parents and providers did not reject AI outright, but emphasised that AI should remain supportive, transparent, and embedded within human care relationships. In Saudi Arabia, studies of public and physician perceptions have reported interest in AI's potential benefits while also identifying concerns related to privacy, safety, accountability, and professional preparedness [43, 44]. Similarly, work from the United Arab Emirates has emphasised the importance of physician involvement, training, workflow integration, and maintaining professional control over AI‐supported care [45]. Evidence from Bahrain also shows that physicians recognise both benefits and drawbacks of AI, reinforcing the need for cautious implementation, professional preparation, and attention to trust [46].

The present study contributes to this emerging regional literature by providing qualitative evidence from rural Iran, a context with a distinctive primary healthcare infrastructure built around the Behvarz community health worker network. Unlike much of the regional literature, which focuses primarily on urban health systems, physicians, or generalised AI readiness, this study foregrounds the perspectives of parents of children with chronic illness and rural healthcare providers. The findings show that AI was valued not as a replacement for clinicians, but as a potential bridge to timely, human‐centred care when specialist access, travel costs, and workforce shortages constrain routine chronic disease management.

The concept of “AI as a bridge to human‐centred care” should therefore be understood as an empirically grounded interpretation from this study rather than as a previously established regional framework. Participants' accounts suggest that AI may be acceptable in rural pediatric chronic care when it extends access to human expertise, supports Behvarzan and clinicians, and preserves relational trust. This framing adds a context‐specific contribution to the Middle Eastern digital health literature while avoiding overgeneralisation across diverse countries and health systems.

#### 4.3.4 Implications for Practice

The findings have several implications for clinical practice in rural healthcare settings. First, healthcare providers should be involved in the design and implementation of AI systems to ensure alignment with clinical workflows and professional values. Second, AI implementations should be positioned as decision support tools that augment rather than replace clinical judgement, with clear mechanisms for human oversight. Third, training programs for both providers and patients should accompany AI implementation to address digital literacy barriers.

The emphasis on embedding AI within existing Behvarz networks provides a practical model for implementation that leverages existing infrastructure rather than requiring entirely new systems. This approach is consistent with global recommendations for task‐shifting and task‐sharing in healthcare delivery.

#### 4.3.5 Health Policy Implications

The findings support several policy recommendations for AI implementation in rural pediatric healthcare:

1. Phased Implementation Strategy: Findings indicate that participants preferred AI as a supplement to rather than replacement of human care. Policies should prioritise AI applications that enhance the capabilities of Behvarzan rather than displacing human contact. Implementation should begin with low‐stakes applications (e.g., appointment scheduling, health education) before advancing to diagnostic support.
2. Infrastructure Investment Prioritisation: Given participants' concerns about internet connectivity and power reliability, policy should address infrastructure prerequisites before AI deployment. The finding that approximately 78% of rural households have internet access underscores the need for continued infrastructure development. This aligns with research identifying infrastructure as a critical determinant of digital health success in low‐ and middle‐income countries.
3. Training Programs for Behvarzan: Participants emphasised the critical role of Behvarzan in rural healthcare delivery. AI implementation policies should include comprehensive training programs that position Behvarzan as AI facilitators, not technology replacements. Digital literacy training has been identified as a critical success factor for health technology adoption in resource‐limited settings.
4. Privacy Governance Frameworks: Parents expressed significant concerns about data privacy, particularly for children with chronic illnesses. Policy should establish clear data governance frameworks that address parental consent, data storage, and third‐party access. This is consistent with international guidelines on health data protection and AI ethics in healthcare.
5. Pilot Programs with Evaluation: Given the anticipated nature of participants' expectations, policy should support pilot programs with rigorous evaluation to assess actual versus perceived feasibility. This approach allows for iterative refinement based on real‐world implementation experience.

### 4.4 Limitations

Several limitations must be acknowledged. First, the qualitative design limits generalisability, and participants' expectations were largely anticipatory rather than based on actual AI experience. Second, the study was conducted in three districts of Isfahan Province, and findings may not be transferable to other regions of Iran with different healthcare infrastructure or cultural characteristics. Third, the cross‐sectional design cannot capture changes in perceptions over time. Fourth, social desirability bias may have influenced participants' responses, particularly regarding sensitive topics such as financial strain and caregiver burden. Fifth, the translation process, while rigorous, may have introduced some loss of nuance in participant quotations.

### 4.5 Future Research Directions

Future research should examine actual AI adoption patterns following implementation, assess the accuracy of participants' expectations against real‐world outcomes, and compare findings across diverse rural contexts within Iran and the broader Middle East region. Such research will contribute to evidence‐based AI implementation strategies that balance technological innovation with the humanistic values central to healthcare delivery.

Specifically, future studies should [1]: conduct quantitative surveys to validate the themes identified in this qualitative study across larger samples [2]; implement pilot AI interventions and evaluate their effectiveness in improving healthcare access [3]; examine the perspectives of children with chronic illnesses themselves, as they were not directly included in this study [5]; investigate the cost‐effectiveness of different AI implementation models in rural settings; and [6] explore the role of gender in shaping AI perceptions among parents and providers.

## 5 Conclusion

This qualitative investigation provides foundational understanding of parent and provider expectations regarding AI as a bridge to human‐centred care for children with chronic illness in rural Iran. The findings reveal a complex landscape of hopeful anticipation tempered by skeptical concerns, shaped by the unique socio‐cultural and infrastructural context of rural healthcare delivery.

The study makes several contributions to the literature. First, it addresses a significant gap by examining AI perceptions among understudied stakeholders' rural community health workers (Behvarzan) and parents of children with chronic illnesses in a Middle Eastern context. Second, it provides empirical evidence that challenges assumptions about technology acceptance in resource‐limited settings, demonstrating that even populations with limited prior AI exposure hold sophisticated and nuanced expectations. Third, it offers empirically grounded policy recommendations for AI implementation that prioritise human‐centred approaches.

The central finding that AI is most likely to be accepted when positioned as a complement to rather than replacement of human care has important implications for technology design and policy. The emphasis on embedding AI within existing Behvarz networks provides a practical model for implementation that leverages existing infrastructure rather than requiring entirely new systems. The call for co‐designing platforms with families ensures that cultural context is central to technology development.

## Author Contributions

**Atefeh Shamsi:** conceptualisation, methodology, investigation, formal analysis, data curation, writing original draft, writing review and editing, visualisation, project administration. **Mahboobeh Namnabati:** methodology, investigation, formal analysis, writing – review and editing. **Asghar Ehteshami:** methodology, resources, validation, writing review and editing. **Hamed Zandi Esfahani:** investigation (clinical context validation), resources (pediatric chronic disease expertise), review clinical implications section. All Authors: read and approval – final manuscript.

## Funding

The authors have nothing to report.

## Ethics Statement

This study was approved by the Ethics Committee of Isfahan University of Medical Sciences (Approval Code: IR.MUI.NUREMA.REC.2022.055). All procedures were conducted in accordance with the ethical standards of the institutional research committee and the 1964 Declaration of Helsinki and its later amendments.

## Consent

Written informed consent was obtained from all individual participants before interview commencement. Participants were informed of their right to withdraw from the study at any time without consequence to their healthcare services. For publication of anonymized direct quotations and aggregated findings, written informed consent was obtained from all participants. All identifying information has been removed or altered to prevent recognition of individuals or specific rural communities. Pseudonyms have been assigned to all quoted participants.

## Conflicts of Interest

The authors declare no conflicts of interest.

## Data Availability

The data that support the findings of this study are available from the corresponding author upon reasonable request.
